# Letter to the Editor: “Environmental impact of current endoscopic technology in urological procedures: a systematic review on reusable vs. disposable scopes”

**DOI:** 10.1007/s00345-025-05454-2

**Published:** 2025-01-22

**Authors:** Marlene Thöne, Steffen Rausch

**Affiliations:** 1 Department of Urology, RWTH University Hospital Aachen, Pauwelsstraße 30, D-52074 Aachen, Germany; 2 Department of Urology, University Hospital Tubingen, Hoppe-Seyler-Str. 3, D-72076 Tubingen, Germany

With great interest, we read the systematic review by Peyrottes and Thibaut Long-Depaquit et al. [1]. and commend the authors for their exceptional work in addressing this important topic. Their detailed analysis and keen awareness of the existing literature are greatly appreciated. In addition, it is particularly inspiring to see a strong team in France dedicated to advancing “urologic sustainability.”

The review provides valuable insights into the environmental impact of reusable versus disposable scopes. The data presented, such as the reduced water and waste associated with single-use cystoscopes compared to reusable devices, highlight the complexities of this issue. The clear delineation of the limitations inherent in the included studies and the emphasis on the urgent need for standardized research and detailed examinations of individual devices are particularly noteworthy.

A recent publication by our group presented a Life Cycle Assessment (LCA) that evaluates the environmental impact of flexible ureterorenoscopes (fURS), comparing single-use and reusable devices. By conducting an LCA in accordance with ISO 14,040/44 at a German university clinic powered by renewable energy, it was determined that single-use fURS have a threefold higher potential environmental impact compared to reusable fURS (4.32 vs. 1.24 kg CO2 equivalents) [2]. Sensitivity analyses, which accounted for variables such as electricity mix and increased water usage for reprocessing reusable devices, consistently favored the reusable option. Notably, the waste generated by a single-use device far exceeded that produced during one procedure with a reusable device.

To enhance the accessibility of these findings, an evaluation of Disability-Adjusted Life Years (DALYs) using the ReCiPe2016(H) method was incorporated, as an approach to make results from LCA more comprehensible for medical professionals and support broader discussions on the environmental impacts of healthcare practices.

In conclusion, the points raised in the present publication are strongly supported, and the data on fURS are offered as a complementary contribution to the ongoing dialogue. Further research in this field is highly encouraged, particularly the replication of the suggested methodological approach across various urological procedures. As a healthcare community, it is essential to deepen the understanding of the environmental footprint of urology and identify the most effective interventions to mitigate it. The evidence is clear: healthcare, including urology, impacts the environment. Collaborative efforts are needed to estimate its scale accurately and address it wisely.

## Data Availability

No datasets were generated or analysed during the current study.

## References

[1] Peyrottes A, Long-Depaquit T, Pradere B et al (2024) Environmental impact of current endoscopic technology in urological procedures: a systematic review on reusable vs. disposable scopes. World J Urol 43(1):15 39638871 10.1007/s00345-024-05317-2

[2] Thone M, Lask J, Hennenlotter J et al (2024) Potential impacts to human health from climate change: a comparative life-cycle assessment of single-use versus reusable devices flexible ureteroscopes. Urolithiasis 52(1):166 39579203 10.1007/s00240-024-01664-2 PMC11585494

